# Volumetric-Modulated Arc-Based Re-radiosurgery With Simultaneous Reduced-Dose Whole-Brain Irradiation for Local Failures Following Prior Radiosurgery of Brain Oligometastases From Small Cell Lung Cancer

**DOI:** 10.7759/cureus.44492

**Published:** 2023-08-31

**Authors:** Kazuhiro Ohtakara, Sosuke Arakawa, Makoto Nakao, Hideki Muramatsu, Kojiro Suzuki

**Affiliations:** 1 Department of Radiation Oncology, Kainan Hospital Aichi Prefectural Welfare Federation of Agricultural Cooperatives, Yatomi, JPN; 2 Department of Radiology, Aichi Medical University, Nagakute, JPN; 3 Department of Respiratory Medicine, Nagoya City University East Medical Center, Nagoya, JPN; 4 Department of Respiratory Medicine, Kainan Hospital Aichi Prefectural Welfare Federation of Agricultural Cooperatives, Yatomi, JPN

**Keywords:** volumetric-modulated arc-based radiosurgery, simultaneous integrated boost, fractionation, biological effective dose, re-irradiation, stereotactic radiosurgery, limited-stage, chemoradiotherapy, small cell lung cancer, brain metastases

## Abstract

First-line and possibly repeated stereotactic radiosurgery (SRS) with preserving whole-brain radiotherapy (WBRT) is an attractive and promising option for synchronous or metachronous limited brain metastases (BMs) from small cell lung cancer (SCLC), for which a modest prescription dose is generally preferred, such as a biological effective dose of ≤60 Gy, based on the linear-quadratic formula with an alpha/beta ratio of 10 (BED_10_). In addition, the optimal planning scheme for re-SRS for local progression after SRS of BMs from SCLC remains unclear. Herein, we describe a case of limited BMs developing after a partial response to standard chemoradiotherapy (CRT) for limited-stage SCLC. The BMs, including local failures following prior single-fraction (fr) SRS, were re-treated with volumetric-modulated arc-based SRS combined with simultaneous reduced-dose WBRT. The first SRS with 36.3 Gy/3 fr (BED_10_ 80 Gy) for a small BM resulted in a local control of 17.2 months. However, the second SRS with 20 Gy/1 fr (BED_10_ 60 Gy) to the 60% or 85% isodose surface (IDS) covering the gross tumor volume (GTV) of three new BMs with a paradoxical T1/T2 mismatch, that is, a visible mass on T2 larger than an enhancing area, resulted in partial symptomatic local progression of all lesions within 5.2 months, along with the development of two new lesions, despite continued amrubicin monotherapy. In contrast, the third SRS with 53 Gy/10 fr (BED_10_ 81 Gy) to ≤74% IDSs encompassing the GTV boundary resulted in complete responses of all the lesions during six months. However, despite a combined use of WBRT of 25 Gy in the third SRS, symptomatic spinal cerebrospinal fluid dissemination and new BMs developed, the former leading to patient mortality. A BED_10_ of ≥80 Gy to the GTV margin and a steep dose increase inside the GTV boundary are suitable to ensure excellent local control in SRS for SCLC BMs. Re-SRS with the aforementioned scheme can be an efficacious option for local failures following prior SRS with a BED_10_ of ≤60 Gy. Modest dose escalation with a simultaneous integrated boost to bulky lesions in the initial CRT may reduce the development of new BM through improved control of the potential source.

## Introduction

Small cell lung cancer (SCLC) has an intrinsically high predisposition to develop brain metastases (BMs), and accurate diagnosis, treatment, and prevention are important for preserving neurocognitive function and quality of life and improving survival in affected patients [1]. Given the limited penetration of systemic therapy into the BMs and the central nervous system (CNS) as a sanctuary site, external beam radiotherapy (EBRT) plays an important role in managing patients with SCLC harboring macroscopic and/or microscopic BMs [1]. Compared to non-small cell lung cancer (NSCLC) or other solid malignancies, whole-brain radiotherapy (WBRT) has traditionally been preferred over stereotactic radiosurgery (SRS) for SCLC BMs, and SRS has been utilized mainly as a salvage treatment for regrowth and/or new BMs following prior therapeutic or prophylactic WBRT [2,3]. Leksell Gamma Knife® (LGK, Elekta AB, Stockholm, Sweden) and general-purpose linacs with volumetric-modulated arcs (VMA) have enabled efficient SRS for more than 5-10 BMs [4]. Modest prescription doses are used commonly and frequently for SRS of SCLC BMs, such as a biologically effective dose of ≤60 Gy, for example, 18-20 Gy in a single fraction (fr), based on the linear-quadratic formula with an alpha/beta ratio of 10 (BED_10_), under the prevailing view of high radiosensitivity of SCLC [2,3]. WBRT is proverbially associated with acute and late detrimental effects on the brain and surrounding tissues, including neurocognitive decline [5]. In addition, WBRT for oligo-BMs is, in principle, a single-use tool for controlling microscopic BMs and/or intracranial extra-parenchymal dissemination present at the initiation of irradiation.

Driven by advances in systemic therapy, up-front and possibly repeated SRS with preservation of WBRT as a last resort has become an attractive and promising option for selected patients with synchronous or metachronous limited BMs from SCLC [1,5-8]. Compared with WBRT with or without SRS, SRS alone is associated with a shorter period for developing new BMs with more lesions; however, with appropriate salvage treatment, the overall survival (OS) after SRS is comparable to that after WBRT [6-8]. Furthermore, a recent comparative study suggested that first-line SRS for SCLC BMs is associated with shorter OS compared to NSCLC; however, neurological mortality, the number of new BMs, and leptomeningeal progression are similar in patients matched for baseline characteristics [9]. As survival improves, the evaluation and salvage treatment for local progression after SRS are becoming more important. However, the optimal planning scheme for re-SRS in these scenarios remains unclear [10].

Herein, we describe a case of limited BM development after a partial response (PR) following concurrent chemoradiotherapy (CCRT) for limited-stage (LS) SCLC, in which SRS with different dose fractions was performed four times for single- or oligo-BMs that developed at different times during two years. In particular, five BMs, including three local failures following prior SRS with 20 Gy/1 fr (BED_10_ 60 Gy), were treated or re-treated with VMA-based SRS using 53 Gy/10 fr (BED_10_ of >80 Gy) combined with simultaneous reduced-dose WBRT. Although excellent local control was achieved, the patient eventually died of cerebrospinal fluid (CSF) dissemination. We specifically discuss the optimal dose and distribution of SRS for SCLC BMs in radiation-naïve and re-irradiation settings and a possible measure to minimize the development of new BMs with subsequent CSF dissemination.

This report was part of the clinical study approved by the Clinical Research Review Board of Kainan Hospital Aichi Prefectural Welfare Federation of Agricultural Cooperatives (20220727-1).

## Case presentation

**Figure 1:**
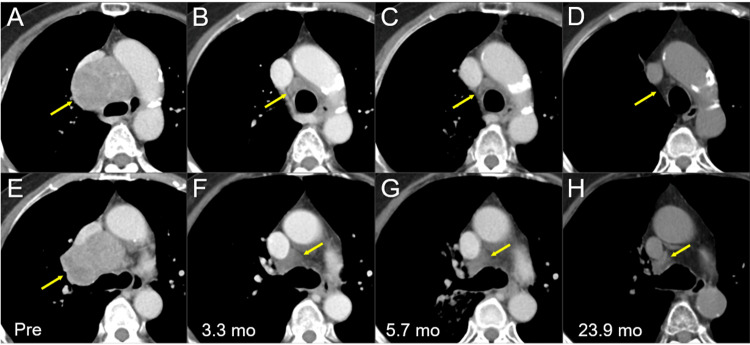
Thoracic computed tomography images before and after chemoradiotherapy. The images show axial (A-C, E-G) CE CT images and axial (D, H) non-CE-CT images (A, E) 28 days before (Pre) the initiation of CRT, (B, F) at 3.3 mos after CRT initiation (after four courses of EC), (C, G) at 5.7 mos (after six courses of EC), and (D, H) at 23.9 mos. (A-H) These images are shown at the same magnification and to some extent the same coordinates under co-registration and partly manual fusions. (A, B) Well-demarcated heterogeneously enhanced mass lesions (arrows in A and E) in the mediastinum and right hilum, with the displacement of the trachea, bilateral bronchi, esophagus, and superior vena cava. (B, C, F, and G) The lesions regressed remarkably, however, still leaving the residual masses (arrows in B, C, F, and G). (D, H)  The residual mass lesions further regressed (arrows in D and H) at 23.9 mos. CE, contrast-enhanced; CT, computed tomography, CRT, chemoradiotherapy; mo, month; EC, etoposide plus cisplatin

A 65-year-old, right-handed female who was an ex-smoker presented with asymptomatic mediastinal mass lesions detected by screening. The clinicopathological examination revealed LS-SCLC (Figure 1).

**Figure 2:**
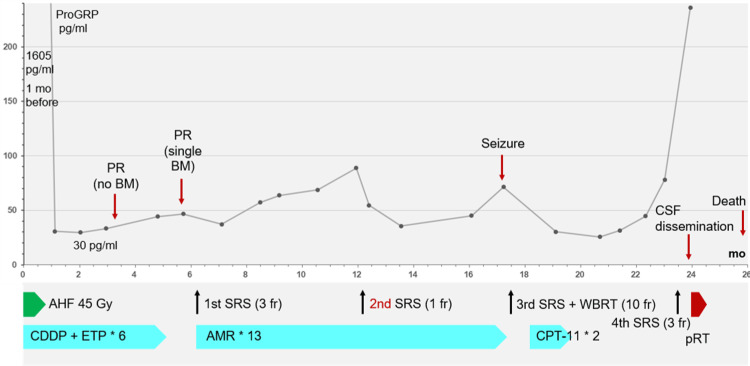
Summary of anticancer treatments along with a series of changes in the ProGRP level. CDDP + ETP: CDDP of 80 mg/m^2^ (day 1) and ETP of 100 mg/m^2^ (days 1-3) with 20% dose reduction from the third course, due to myelotoxicity; AMR monotherapy: AMR of 35 mg/m^2^ (days 1-3) with dose reduction of 30 mg/m^2^ from the second course, due to myelotoxicity; CPT-11 monotherapy: CPT-11 of 80 mg/m^2^ (days 1, 8, and 15) with dose reduction of 60 mg/m^2^ from the second course, partly due to a compound heterozygous carrier with the *6/*28 allele variants of the *UGT1A1* gene. ProGRP, pro-gastrin-releasing peptide; mo, month; PR, partial response; BM, brain metastasis; CSF, cerebrospinal fluid; AHF, accelerated hyperfractionation; SRS, stereotactic radiosurgery; fr, fraction; WBRT, whole-brain radiotherapy; CDDP, cisplatin; ETP, etoposide; AMR, amrubicin; CPT-11, irinotecan; pRT, palliative RT for symptomatic spinal dissemination; UGT1A1, uridine disphosphate glucuronosyltransferase 1

The anticancer treatments are summarized in Figure 2.

The patient initially received standard CCRT consisting of etoposide and cisplatin (EC) and accelerated hyperfractionation (AHF) of 45 Gy in 30 fr bis in die (BID) [1,11]. The AHF with a 10 megavoltage (MV) X-ray beam was delivered under image guidance with opposed two fields of initial anterior-posterior and subsequent response-adaptive oblique off-cord beams, that is, two-dimensional irradiation techniques using three-dimensional planning by the predecessor.

**Figure 3:**
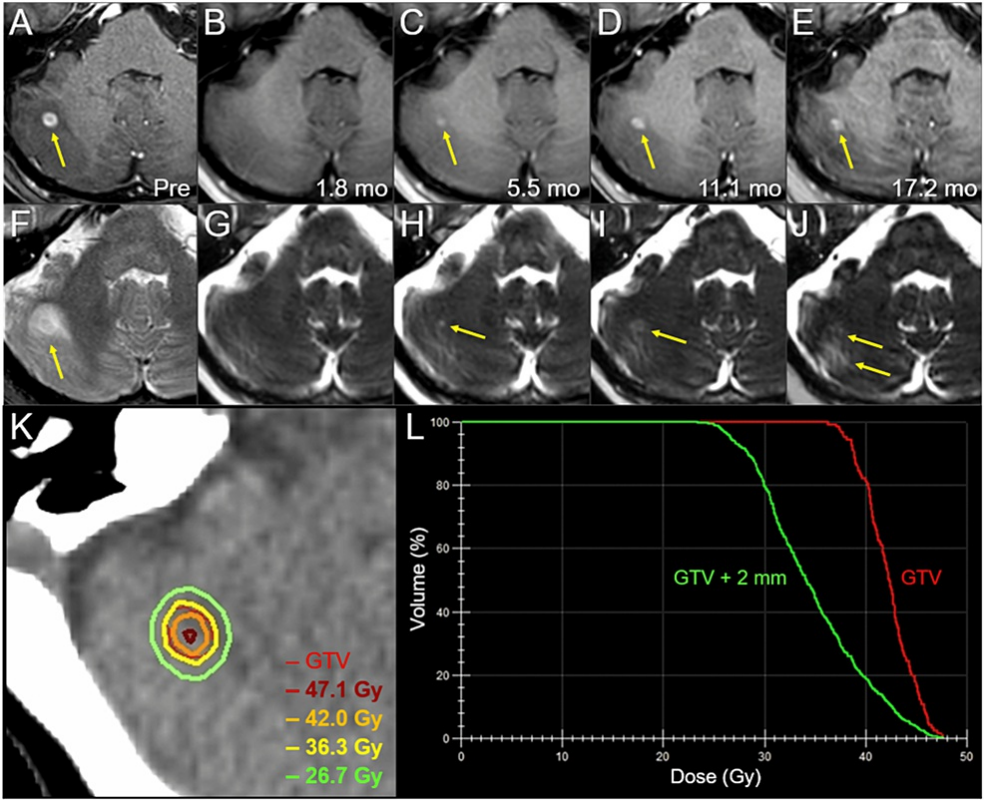
Magnetic resonance images, dose distribution, and dose-volume histograms before and after the first three-fraction stereotactic radiosurgery in the right cerebellar lesion. The images show (A-E) axial CE-T1-WIs and (F-J) axial T2-WIs (A, F) five days before (Pre) the initiation of SRS, (B, G) at 1.8 mos after SRS initiation, (C, H) at 5.5 mos, (D, I) at 11.1 mos, and (E, J) at 17.2 mos; (K) an axial dose distribution; and (L) DVHs. (A-J) These images are shown at the same magnification and coordinates under co-registration and fusions. (A, F) A solid enhancing lesion (arrow in A) with the corresponding mass on T2-WI (T1/T2 match) associated with perilesional edema (arrow in F) in the right cerebellar hemisphere. (B, G) At 1.8 mos, the lesion regressed completely. (C-E, H-J) The enhancing effect gradually increased (arrows in C-E); however, the corresponding mass on T2-WI remained obscure (T1/T2 mismatch) and the perilesional edema was mild (arrows in H-J). (K) The GTV contour (thin line) and representative IDLs (thick lines) are superimposed onto an axial CT image. (L) The GTV + 2 mm structure was generated by adding an isotropic 2-mm margin to the GTV for the evaluation of the dose gradient outside the GTV boundary. CE, contrast-enhanced; WIs, weighted images; SRS, stereotactic radiosurgery; CT, computed tomography; DVH, dose-volume histogram; mo, month; GTV, gross tumor volume; IDL, isodose line

The pro-gastrin-releasing peptide (ProGRP) level decreased to 30 pg/mL at 2.1 months following CCRT (Figure 2), and the thoracic lesions showed PRs after four courses of the EC regimen (3.3 months after the initiation of CCRT) (Figure 1). Prophylactic cranial irradiation (PCI) was not administered, and two additional courses of the EC regimen were administered. The thoracic lesions sustained PRs 5.7 months after CCRT (after six courses of the EC regimen) (Figure 1); however, magnetic resonance imaging (MRI) of the brain revealed a small cerebellar BM (Figure 3).

For refractory relapse, SRS of the BM and second-line amrubicin (AMR) monotherapy were initiated 6.2 months after CCRT (Figure 2). The patient preferred SRS alone to SRS plus reduced-dose WBRT, which has been proposed as an alternative treatment option. The BM was treated with 3-fr SRS, and the gross tumor volume (GTV) was covered with a 76.1% isodose surface (IDS) of 36.3 Gy (Figure 3; Table 1).

**Table 1:** Tumor characteristics and planning parameters for the first 3-fr and the third 10-fr stereotactic radiosurgery with the biological effective dose of ≥80 Gy. *Dosimetric goals of treatment planning. **The BED_10_ for absolute doses of 36.3 Gy in 3 fr, 53 Gy in 10 fr, 26.7 Gy in 3 fr, and 42.5 Gy in 10 fr are 80.2 Gy, 81.1 Gy, 50.5 Gy, and 60.6 Gy, respectively. ***The %IDS of *D*_98%_, relative to *D*_max_. SRS, stereotactic radiosurgery; fr, fraction; Rt, right; GTV, gross tumor volume; *D*_max_, maximum dose; BED_10_, a biologically effective dose based on the linear-quadratic formula with an alpha/beta ratio of 10; *D*_98%_, a minimum dose encompassing at least 98% of the target volume; IDS, isodose surface

Session			First	Third				
Fraction			3 fr	10 fr (re-SRS)	10 fr (re-SRS)	10 fr (re-SRS)	10 fr	10 fr
Tumor location			Rt cerebellar	Lt parietal	Rt frontal	Rt cerebellar	Lt temporal	Rt cerebellar
GTV	Volume		0.13 cm^3^	1.24 cm^3^	0.69 cm^3^	1.02 cm^3^	1.55 cm^3^	1.69 cm^3^
	*D*_max_ (BED_10_)	BED_10_ ≥120 Gy*	47.7 Gy (123.5 Gy)	74.4 Gy (129.8 Gy)	72.0 Gy (123.8 Gy)	73.0 Gy (126.3 Gy)	76.7 Gy (135.5 Gy)	75.6 Gy (132.8 Gy)
	BED_10_ 80 Gy: Dose (coverage)**	≥98%*	36.3 Gy (99.6%)	53 Gy (98.9%)	53 Gy (99.4%)	53 Gy (97.4%)	53 Gy (99.0%)	53 Gy (99.2%)
	*D*_98%_ (BED_10_)	BED_10_ ≥80 Gy*	37.4 Gy (84.0 Gy)	53.3 Gy (81.7 Gy)	54.0 Gy (83.2 Gy)	52.8 Gy (80.7 Gy)	53.8 Gy (82.7 Gy)	53.8 Gy (82.7 Gy)
	%IDS***	≤77/75% (3 fr/10 fr)*	78.4%	71.6%	75.0%	72.3%	70.1%	71.2%
GTV + 2 mm	*D*_98%_ (BED_10_)	BED_10_ ≥50/60 Gy (3 fr/10 fr)*	25.7 Gy (47.7 Gy)	43.0 Gy (61.5 Gy)	44.5 Gy (64.3 Gy)	43.6 Gy (62.6 Gy)	43.9 Gy (63.2 Gy)	44.9 Gy (65.1 Gy)
	BED_10_ 50/60 Gy: Dose (coverage)**	≥95%*	26.7 Gy (95.0%)	42.5 Gy (98.8%)	42.5 Gy (99.8%)	42.5 Gy (99.3%)	42.5 Gy (99.7%)	42.5 Gy (99.9%)

The SRS was implemented with VMA using a multileaf collimator Agility® (Elekta AB) mounted in a linac Infinity® (Elekta AB) using a flattening filter-free mode of a 6 MV X-ray beam [4,12]. The dedicated software MIM Maestro^TM^ (MIM Software, Cleveland, OH, USA) was used for image co-registration and contouring [10]. The VMA optimization was performed with a planning system Monaco® (Elekta AB). The arc arrangement consisted of one coplanar arc and two noncoplanar arcs, which were allocated at 60º intervals to divide the cranial hemisphere evenly [12]. MRI performed 1.8 months after SRS showed a complete response (CR) of the BM (Figure 3). Although AMR monotherapy was continued, the ProGRP level gradually increased and exceeded the upper limit of the normal range 12 months after CCRT (Figure 2).

**Figure 4:**
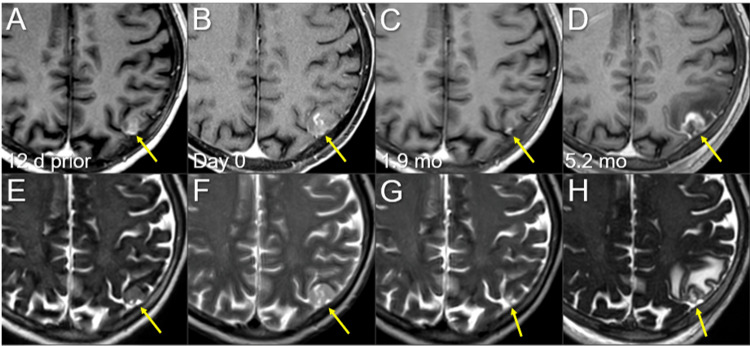
Magnetic resonance images before and after the second single-fraction radiosurgery of the left parietal lesion. The images show (A-D) axial CE-T1-WIs and (E-H) axial T2-WIs (A, E) 12 days before (12 d prior) the second SRS (11.8 mos after the initiation of CRT); (B, F) on the day of SRS (day 0); (C, G) at 1.9 mos after the second SRS; and (D, H) at 5.2 mos (17.3 mos after CRT). (A-H) These images are shown at the same magnification and coordinates under co-registration and fusions. (A, E) A heterogeneously enhancing lesion (arrow in A) without perilesional edema (arrow in E). (B, F) Notably, the lesion increased in size with higher intensity on T2-WIs (arrows in E and F) in 12 days, and the contrast enhancement is faint and partial (arrow in B) compared to the visible mass on  T2-WI (T2-mass, arrow in F), that is, a paradoxical T1/T2 mismatch. Furthermore, the main location of the lesion is the brain surface, the cerebral cortex, with partly exophytic growth beyond the brain surface and attachment to the adjacent dura mater. (C, G) At 1.9 mos, the lesion shrank remarkably (arrows in C and G). (D, H) The enhancing lesion and T2-mass obviously increased (arrows in D and H), along with the development of perilesional edema. CE, contrast-enhanced; WIs, weighted images; SRS, stereotactic radiosurgery; CRT, chemoradiotherapy; mo, month

**Figure 5:**
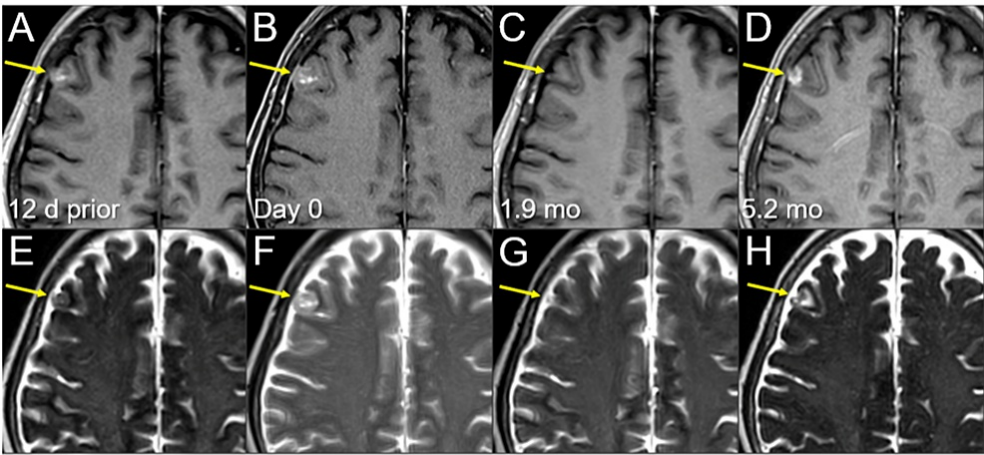
Magnetic resonance images before and after the second single-fraction radiosurgery of the right frontal lesion. The images show (A-D) axial CE-T1-WIs and (E-H) axial T2-WIs (A, E) 12 days before (12 d prior) the second SRS (11.8 months after the initiation of CRT); (B, F) on the day of SRS (day 0); (C, G) at 1.9 mos after the second SRS; and (D, H) at 5.2 months (17.3 months after CRT). (A-H) These images are shown at the same magnification and coordinates under co-registration and fusions. (A, E) A heterogeneously enhancing lesion (arrow in A) without perilesional edema (arrow in E). (B, F) The lesion increased in size with higher intensity on T2-WIs (arrows in E and F) in 12 days, and the contrast enhancement is faint and partial (arrow in B) compared to the visible mass on  T2-WI (T2 mass, arrow in F), a paradoxical T1/T2 mismatch. Furthermore, the main location of the lesion is the brain surface, the cerebral cortex, with partly exophytic growth beyond the brain surface and attachment to the adjacent dura mater. (C, G) At 1.9 mos, the lesion shrank remarkably (arrows in C and G). (D, H) The enhancing lesion and T2 mass obviously increased (arrows in D and H), along with the development of perilesional edema. CE, contrast-enhanced; WIs, weighted images; SRS, stereotactic radiosurgery; CRT, chemoradiotherapy; T2 mass, a visible mass on T2-WIs; mo, month

**Figure 6:**
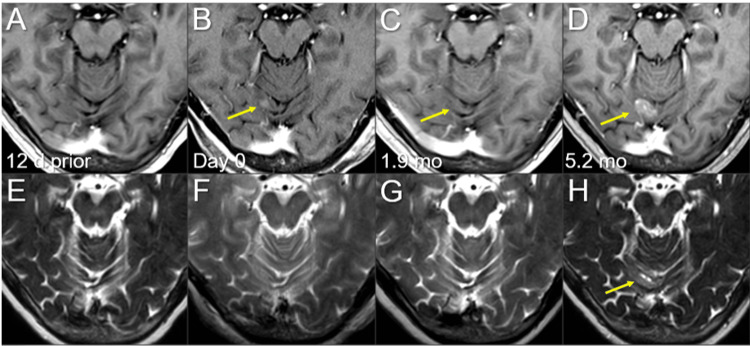
Magnetic resonance images before and after the second single-fraction radiosurgery of the right cerebellar lesion. The images show (A-D) axial CE-T1-WIs and (E-H) axial T2-WIs (A, E) 12 days before (12 d prior) the second SRS (11.8 mos after the initiation of CRT); (B, F) on the day of SRS (day 0); (C, G) at 1.9 mos after the second SRS; and (D, H) at 5.2 mos (17.3 mos after CRT). (A-H) These images are shown at the same magnification and coordinates under co-registration and fusions. (A, B, E, F) A solid enhancing lesion (arrow in B) appeared in 12 days. (C, G) At 1.9 mos, the lesion almost disappeared (arrow in C). (D, H) The enhancing lesion increased remarkably beyond its original size, along with the T2 mass (arrows in D and H). CE, contrast-enhanced; WIs, weighted images; SRS, stereotactic radiosurgery; CRT, chemoradiotherapy; T2 mass, a visible mass on T2-WIs; mo, month

MRI revealed three new superficial BMs (Figures 4-6) and a reappearance of the enhancing effect in the initial right cerebellar lesion (Figure 3).

**Figure 7:**
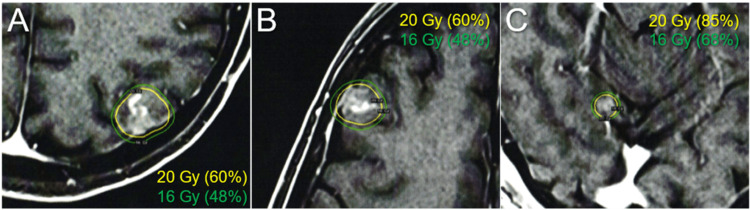
Dose distributions of the second single-fraction stereotactic radiosurgery. The images show representative IDLs (solid lines) superimposed onto (A-C) axial CE-T1-WIs: (A) the left parietal lesion; (B) the right frontal lesion; and (C) the right cerebellar lesion. (A-C) The percentage value in the parentheses following the absolute dose is the percentage of isodose relative to the maximum dose. The GTVs defined based on the visible masses on T2-WIs are conformally covered with the 20 Gy IDLs. The dose gradients from 20 to 16 Gy are generally steep in all the lesions. IDLs, isodose lines; CE, contrast-enhanced; WIs, weighted images; GTVs, gross tumor volumes

The three new BMs were treated with a second SRS alone at 20 Gy/1 fr using LGK (Elekta AB) at another facility at the patient’s and family’s request (Figure 7; Table 2).

**Table 2:** Tumor characteristics and planning parameters for the second single-fraction stereotactic radiosurgery with 20 Gy. *The %IDSs represent a uniform prescription dose of 20 Gy relative to *D*_max_ (100%). **The number of shots (isocenters) and the collimator sizes and combinations in the parentheses. Lt, left; Rt, right; GTV, gross tumor volume; IDS, isodose surface; *D*_max_, maximum dose

Tumor location	Lt parietal	Rt frontal	Rt cerebellar
GTV (cm^3^)	2.19	1.39	0.20
GTV marginal dose (Gy)	20	20	20
%IDS*	60%	60%	85%
*D*_max _(Gy)	33.3	33.3	23.5
Shot(s) (collimator)	17 shots (8 and 16 mm)	12 shots (8 and 16 mm)	1 shot (8 mm)

**Figure 8:**
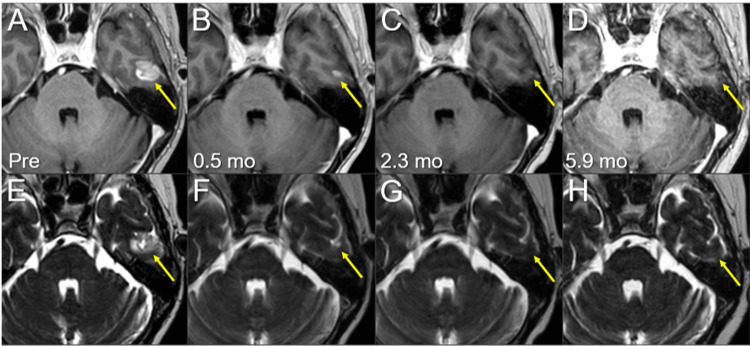
Magnetic resonance images before and after the third SRS of the left temporal new lesion. The images show (A-D) axial CE-T1-WIs and (E-H) axial T2-WIs (A, E) five days before (Pre) the third SRS (17.3 mos after the initiation of CRT); (B, F) at 0.5 mo after the initiation of the third SRS; (C, G) at 2.3 mos; and (D, H) at 5.9 mos (23.9 mos after CRT). (A-H) These images are shown at the same magnification and coordinates under co-registration and fusions. (A-H) The solid enhancing lesion (arrows in A and E) shrank remarkably at 0.5 mo (arrows in B and F) and disappeared at 2.3 mos (arrows in C and G). The lesion remained completely regressed without adverse radiation effects at 5.9 mos (arrows in D and H). CE, contrast-enhanced; WIs, weighted images; SRS, stereotactic radiosurgery; CRT, chemoradiotherapy; mo, month

**Figure 9:**
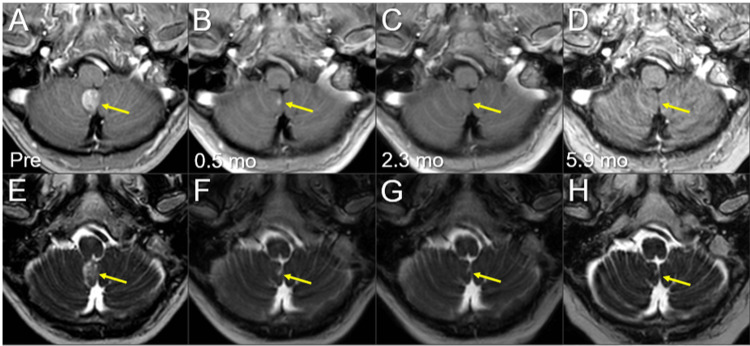
Magnetic resonance images before and after the third SRS of the right cerebellar new lesion. The images show (A-D) axial CE-T1-WIs and (E-H) axial T2-WIs (A, E) five days before (Pre) the third SRS (17.3 mos after the initiation of CRT); (B, F) at 0.5 mo after the initiation of the third SRS; (C, G) at 2.3 mos; and (D, H) at 5.9 mos (23.9 mos after CRT). (A-P) These images are shown at the same magnification and coordinates under co-registration and fusions. (A-H) The solid enhancing lesion (arrows in A and E) shrank remarkably at 0.5 mo (arrows in B, F) and disappeared, only leaving the faint scar (arrow in G), at 2.3 mos (arrows in C and G). The lesion remained completely regressed without any adverse radiation effects at 5.9 mos (arrows in D and H). CE, contrast-enhanced; WIs, weighted images; SRS, stereotactic radiosurgery; CRT, chemoradiotherapy; mo, month

Notably, all three lesions increased during the 12 days before SRS (Figures 4-6). In addition, in the two large lesions, the mass visible on T2-weighted images (WIs) was larger than the enhancing area, indicating a paradoxical T1/T2 mismatch (Figures 4, 5). The thoracic lesions sustained a PR, and AMR monotherapy was continued (Figure 2). MRI at 1.9 months after the second SRS showed marked regression of all three lesions (Figures 4-6). However, the patient developed an epileptic seizure during the 13th course of AMR therapy (5.1 months after the second SRS). MRI revealed obvious local progression of all three lesions treated with the second SRS (Figures 4-6) and two new lesions (Figures 8, 9).

**Figure 10:**
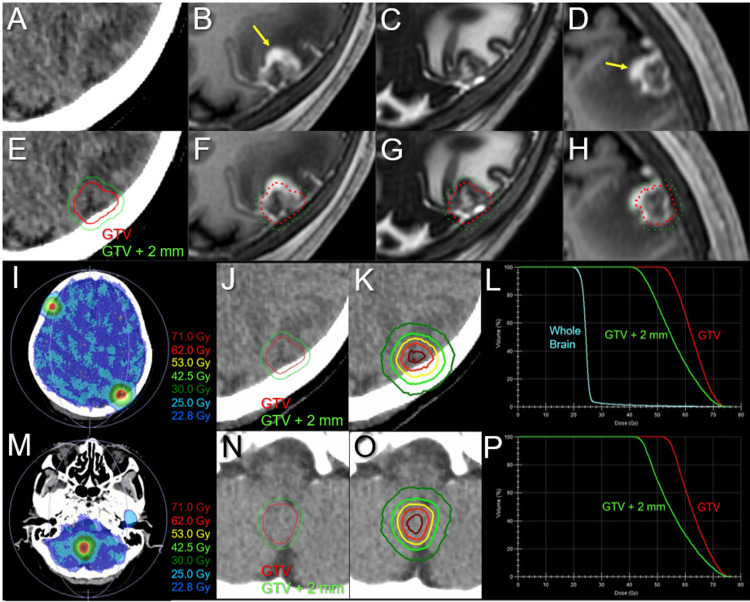
Target definitions, dose distributions, and dose-volume histograms for the third 10-fr stereotactic radiosurgery combined with simultaneous whole-brain irradiation. The images show (A, E) non-CE-CT images and (B, D, F, H) CE-T1-WIs, (C, G) T2-WIs, (I, K, M, O) dose distributions, (E-H, J, N) target definitions, (L, P) DVHs, (A-C, E-G, I-K, M-O) axial images, (D, H) sagittal images, (A-L) the left parietal lesion with local progression following prior SRS, and (M-P) the new right cerebellar lesion. (A-H) These images are shown at the same magnification and coordinates under co-registration and fusions. (A-H) The GTV was defined, mainly based on the visible mass on the T2-WIs (C, G), with the exclusion of excessive exudation of contrast media (arrows in B and D) by contrasting CE-T1-WIs and T2-WIs (T1/T2 matching). (E, J, N) The GTVs and isotropic 2-mm margin-added structures. (I, K, M, O) The representative isofill doses (I, M) and IDLs (K, O) superimposed onto non-CE-CT images. (I, L, M) *D*_98%_ and *D*_50%_ of the whole brain are 22.8 and 24.5 Gy, respectively. CE, contrast-enhanced; CT, computed tomography; WIs, weighted images; DVHs, dose-volume histograms; SRS, stereotactic radiosurgery; GTV, gross tumor volume; IDLs, isodose lines; *D*_*X*%_, a minimum dose encompassing at least *X*% of the target volume; fr, fraction

All three lesions of local progression following the second SRS were deemed dominant viable tumors because the GTV marginal doses were 20 Gy with a steep dose gradient outside the GTV boundary in the three lesions, along with a homogeneous GTV dose with 85% IDS covering the right cerebellar lesion (Figure 7; Table 2). Three regrowth lesions and two new lesions were treated with 10-fr SRS combined with simultaneous WBRT without hippocampal avoidance to ensure the control of potential meningeal and/or cerebrospinal fluid (CSF) dissemination (Figure 10).

**Figure 11:**
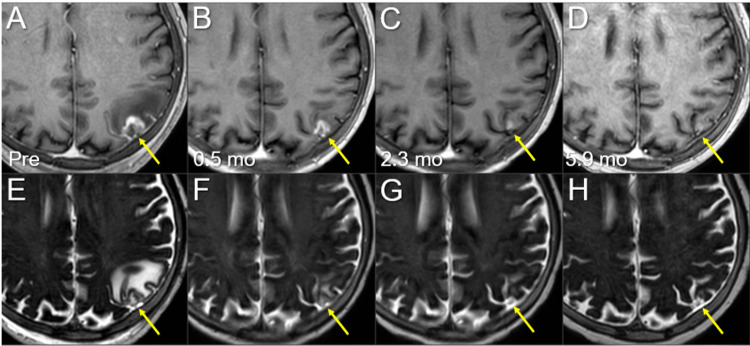
Magnetic resonance images before and after the third 10-fr SRS combined with simultaneous whole-brain irradiation for local failure following prior SRS of the left parietal lesion. The images show (A-D) axial CE-T1-WIs and (E-H) axial T2-WIs (A, E) five days before (Pre) the third SRS (17.3 months after the initiation of CRT); (B, F) at 0.5 mo after the third SRS; (C, G) at 2.3 mos; and (D, H) at 5.9 months after SRS (23.9 mos after CRT). (A-H) These images are shown at the same magnification and coordinates under co-registration and fusions. (A-H) The solid enhancing lesion (arrows in A and E) shrank remarkably from 0.5 to 2.3 mos (arrows in B, C, F, and G) and almost disappeared at 5.9 mos (arrows in D and H). Despite the re-irradiation setting, the pre-existing perilesional edema (arrow in E) disappeared without the development of any adverse radiation effects (arrow in H) at 5.9 mos. CE, contrast-enhanced; WIs, weighted images; SRS, stereotactic radiosurgery; CRT, chemoradiotherapy; mo, month; fr, fraction

**Figure 12:**
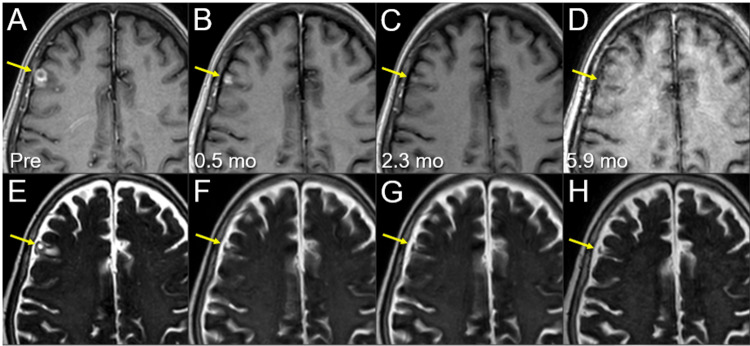
Magnetic resonance images before and after the third 10-fr SRS combined with simultaneous whole-brain irradiation for local failure following prior SRS of the right frontal lesion. The images show (A-D) axial CE-T1-WIs and (E-H) axial T2-WIs (A, E) five days before (Pre) the third SRS (17.3 months after the initiation of CRT); (B, F) at 0.5 mo after the third SRS; (C, G) at 2.3 months; and (D, H) at 5.9 months after SRS (23.9 months after CRT). (A-H) These images are shown at the same magnification and coordinates under co-registration and fusions. (A-H) The solid enhancing lesion (arrows in A and E) shrank remarkably from 0.5 to 2.3 mos (arrows in B, C, F, and G) and almost disappeared at 5.9 mos (arrows in D and H). The pre-existing perilesional edema (arrow in E) disappeared without the development of any adverse radiation effects (arrow in H) at 5.9 mos. CE, contrast-enhanced; WIs, weighted images; SRS, stereotactic radiosurgery; CRT, chemoradiotherapy; mo, month; fr, fraction

**Figure 13:**
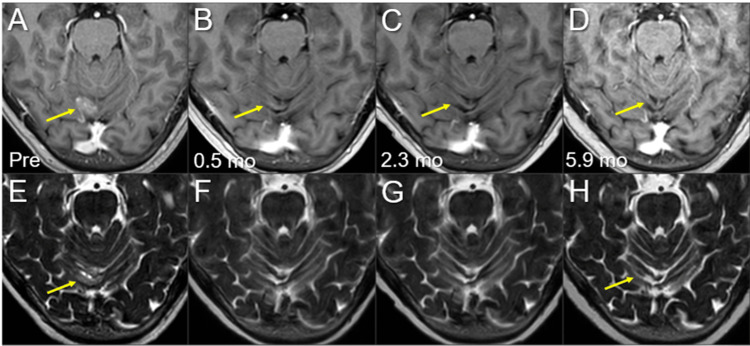
Magnetic resonance images before and after the third 10-fr SRS combined with simultaneous whole-brain irradiation for local failure following prior SRS of the right cerebellar lesion. The images show (A-D) axial CE-T1-WIs and (E-H) axial T2-WIs (A, E) five days before (Pre) the third SRS (17.3 mos after the initiation of CRT); (B, F) at 0.5 mos after the third SRS; (C, G) at 2.3 mos; and (D, H) at 5.9 mos after SRS (23.9 mos after CRT). (A-H) These images are shown at the same magnification and coordinates under co-registration and fusions. (A-H) The solid enhancing lesion (arrows in A and E) shrank remarkably and almost disappeared at 0.5 mos (arrows in B and F). The lesion remained completely regressed without any adverse radiation effects (arrows in C, D, G, and H) at 5.9 mos. CE, contrast-enhanced; WIs, weighted images; SRS, stereotactic radiosurgery; CRT, chemoradiotherapy; mo, month; fr, fraction

GTV contouring for re-SRS was based mainly on mass lesions visible on T2-WI, excluding excessive exudation of contrast material in the surrounding brain (Figure 10). Considering the patient’s concerns regarding radiation-induced cognitive impairment, the WBRT dose was equivalent to that used for PCI. T2-WIs nine days after initiating the third SRS (at 8 fr) showed obvious regression in all five lesions (data not shown). Irinotecan monotherapy instead of AMR was initiated eight days after completing the third SRS; however, it was discontinued after two courses because of severe malaise (Figure 2). At 5.5 months after the third SRS, the ProGRP level increased precipitously (Figure 2), and MRI revealed two new superficial BMs in the right temporal lobe (0.76 cm^3^) and left cerebellar hemisphere (0.40 cm^3^). The initial cerebellar lesion left the enhancing effect dominated by the radiation effect (Figure 3), and the five lesions treated with the second and third SRS remained in CRs (Figures 8, 9, 11-13).

The two new lesions were treated with a fourth 3-fr SRS using a planning scheme similar to that of the first SRS. After completing the fourth SRS, the patient developed unsteadiness and subsequent pain and weakness in the left lower extremity. MRI revealed intradural dissemination in the lumbosacral spine (data not shown), for which palliative EBRT was administered, leading to pain alleviation.

Although the thoracic lesions regressed (Figure 1), the patient developed persistent and progressive headaches during the palliative EBRT and gradually experienced difficulty with oral intake (Figure 2). The patient died of CSF dissemination 25.8 months after CCRT and 19.6 months after the first SRS.

## Discussion

**Figure 14:**
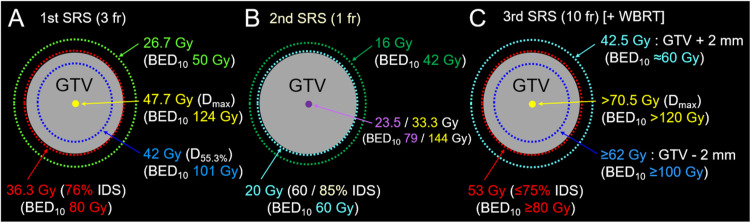
Differences in the planning schemes of the first to third SRS. The schemas show (A-C) a GTV covered by the IDS for dose prescription and the dose gradient outside and inside the GTV boundary: (A) the first 3-fr SRS; (B) the second 1-fr SRS; and (C) the third 10-fr SRS with simultaneous reduced-dose whole brain irradiation. (A-C) The percentage of an IDS is normalized to 100% at *D*_max_. (A) Our basic planning scheme includes a GTV and 2 mm outside the GTV boundary covered with BED_10_s of ≥80 Gy and ≥50 Gy, respectively, and the extremely inhomogeneous GTV dose with concentrically-laminated steep dose increase inside the GTV boundary. By increasing the central dose with the BED10 of ≥120 Gy, the 2 mm inside the GTV boundary is covered with a BED_10_ of ≥100 Gy. (B) The BED_10_s from the GTV boundary to the outside are the lowest. Additionally, in the 85% IDS covering, even the GTV center dose is 23.5 Gy (BED_10_ <80 Gy). (C) In the 10-fr SRS combined with WBRT, the 2 mm outside the GTV boundary was covered with a BED_10_ of ≥60 Gy, a relatively gradual dose gradient, to ensure sufficient coverage of possible microscopic infiltration into the surrounding brain parenchyma. SRS, stereotactic radiosurgery; fr, fraction; WBRT, whole-brain radiotherapy; GTV, gross tumor volume; BED_10_, a biologically effective dose based on the linear-quadratic formula with an alpha/beta ratio of 10; *D*_max_, maximum dose; IDS, isodose surface

With the aging of affected patients, as typified in Japan, strategies centered on SRS and appropriate MRI surveillance in concert with systemic therapy have become even more important in managing patients with BMs from SCLC, especially when the number of BMs remains low. In the present case, single or oligo metachronous BMs developed at intervals of five months or more following the initiation of CCRT: one, three, two, and two new BMs at six, 12, 17, and 23 months, respectively. The two lesions at 12 months differed in paradoxical T2/T2 mismatch from those at other time points. This finding is observed sporadically in some BMs from solid malignancies, including SCLC and NSCLC, and primary CNS lymphoma, suggesting that T2-WIs should be included in MRI for target definition [4,10,13]. PCI or WBRT combined with SRS within six to 12 months of CCRT does not necessarily prevent the development of new BMs 17-23 months after CCRT. Therefore, considering the patient’s preference, applying SRS alone to BMs in the first year was not inappropriate. The differences in the planning schemes of the first to third SRS are shown in Figure 14.

All three BMs treated with the second SRS resulted in partly symptomatic regrowth within 5.2 months; the first and third SRS resulted in better and more durable local control, even in the re-irradiation setting. The dominance of viable tumors at local progression after prior SRS was confirmed by favorable tumor responses after re-SRS (Figure 8). In addition, a planning scheme with adequate consideration of brain tolerance in the second SRS suggests an extremely low probability of brain radionecrosis [14]. In the second SRS, conformal dose distributions with a steep dose gradient outside the GTV boundary were generated by multi-shot planning using the latest planning system. However, the BED_10_ of 20 Gy/1 fr that was the GTV marginal dose in the second SRS corresponds to 60 Gy; the GTV boundary was sufficiently covered with a BED_10_ of ≥80 Gy in the first and third SRS. In general, 24 Gy/1 fr (BED_10_ 81.6 Gy) can yield a 95% one-year local tumor control probability in BMs if ≥98% of a GTV is covered with 24 Gy [14]. In our experience, for 15 years before 2017, many SCLC BMs treated with SRS of ≤18 to 20 Gy/1 fr or a BED_10_ of ≤60 Gy in multiple fractions showed local progression within 0.5 to one year, where 0 to 1 mm outside the GTV boundary was covered with the prescription dose. Therefore, from 2018 onwards, the GTV has been covered with a BED_10_ of ≥80 Gy in SCLC BMs [13]. Furthermore, the relatively homogeneous GTV dose in the second SRS, that is, 85% IDS coverage, was also likely the cause of regrowth, even in the small lesion deemed easily controllable. Thus, extremely inhomogeneous GTV dose, specifically, a concentrically laminated steep dose increase inside the GTV boundary, is an integral part of optimal SRS dose distribution to ensure excellent local control [14,15].

BMs from SCLC or melanoma are characterized by frequent and profound microscopic invasion of the surrounding brain [12,14,16], and a steep dose gradient outside the GTV boundary can compromise sufficient coverage of the brain invasion. Furthermore, a frame-based setup with the potential residual error of a mean value of 0.8 ± 0.4 mm in the LGK could be subjected to further correction by image guidance using cone-beam computed tomography [17]. Thus, an appropriate (not too precipitous or gradual) dose spillage margin outside the GTV boundary and the minimum dose required for tumor control, for example, BED_10_ ≥50 Gy to approximately 2 mm outside the GTV boundary, are additional important elements in SRS for addressing the inherent uncertainties regarding irradiation accuracy [14,18].

The tumor responses after the second SRS suggested that remarkable tumor shrinkage within a few months does not necessarily guarantee complete tumor necrosis and that a partially, not nearly complete, regressed lesion as the maximum response following SRS substantially leaves the probability of a viable tumor [15]. Although the dose gradient inside and outside the GTV boundary and dose fractionation in SRS vary substantially among facilities [19], a dose distribution that achieves a high CR rate or near CR should be determined.

Although the development of new lesions outside the irradiated sites or the subsequent growth and visualization of microscopic lesions present at the time of SRS are unavoidable and within preparedness, preventing or attenuating the development of new BMs by enhancing treatment efficacy to potential sources seems to be another important and potentially manageable issue. Although further advances in systemic therapy are most promising, improving locoregional control in the thorax could be a potential solution, especially in cases of extracranial active disease confined to the thorax, as in the present case. Modest dose escalation with a simultaneous integrated boost to the thoracic lesions, particularly a bulky tumor, can increase the CR rate and may lead to eliminating the source of hematogenous metastases without increasing radiation-induced lung injury [20].

Taken together, the differences in a BED_10_ encompassing a GTV and the dose gradient inside and outside the GTV boundary were shown to yield significant differences in therapeutic effects within six months in the present six lesions. However, the lack of imaging findings more than six months after re-SRS precludes any conclusions regarding its long-term durability and safety. Nevertheless, the present case warrants further investigation to determine the long-term efficacy and safety of the BED_10_-based planning scheme for SRS of SCLC BMs in the first-line and re-irradiation settings.

## Conclusions

When applying SRS alone to oligo-BMs from SCLC, sufficient GTV coverage with a BED_10_ of ≥80 Gy, steep dose increase inside the GTV boundary, and appropriate dose spillage margin outside the GTV are important elements for ensuring excellent local control and preventing CSF dissemination. In the target definition, T2-WIs should be included in the images for contouring to avoid overlooking potential paradoxical T1/T2 mismatches. Re-SRS with the above planning scheme and adequate fractionation can be an efficacious treatment option for local failures following prior SRS with a BED_10_ of ≤60 Gy. Modest dose escalation with a simultaneous integrated boost to bulky lesions in the initial CCRT may attenuate the development of new BMs by eliminating potential sources of hematogenous metastases.
